# Supplementary material for: Exploring the Formation of Polymers with Anti-Amyloid Properties within the 2′3′-Dihydroxyflavone Autoxidation Process
Source: Antioxidants (Basel). 2022 Aug 30;11(9):1711. doi: 10.3390/antiox11091711 (PMC9495709; doi:10.3390/antiox11091711)
Supplement: Supplementary file 1 [file antioxidants-11-01711-s001.zip › antioxidants-1853563-supplementary.pdf]

Exploring the Formation of Polymers with Anti-Amyloid Properties within the 2′3′-Dihydroxyflavone Autoxidation Process

Andrius Sakalauskas, Agne Janoniene, Gediminas Zvinys, Kamile Mikalauskaite, Mantas Ziaunys and Vytautas Smirnovas *

Institute of Biotechnology, Life Sciences Center, Vilnius University, Vilnius, Lithuania

To whom correspondence should be addressed:
Vytautas Smirnovas
Vytautas@smirnovas.info

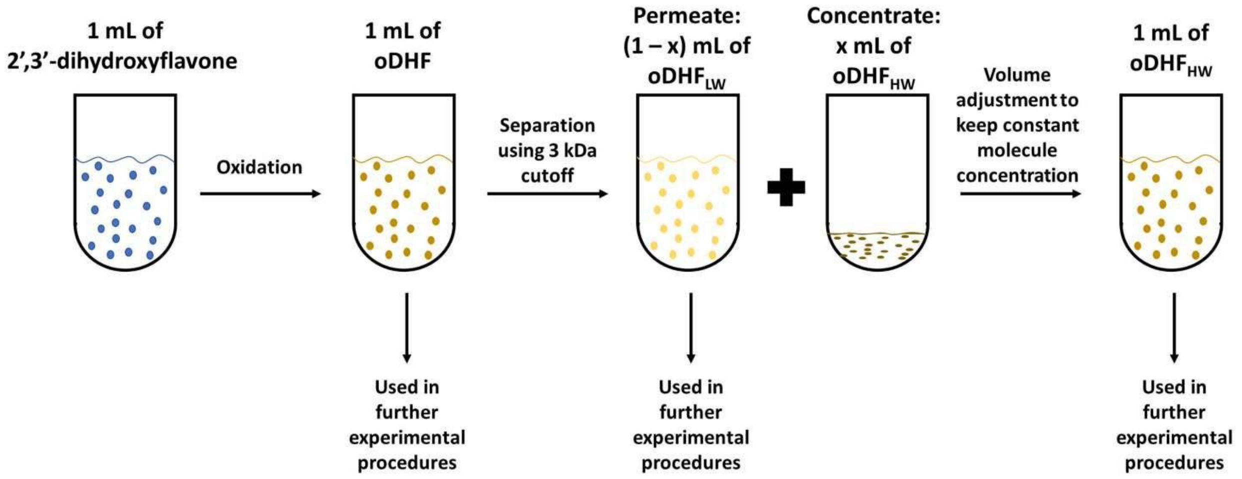

Figure S1. Oxidation of 2',3'-dihydroxyflavone and the separation procedure of the oxidation mixture.

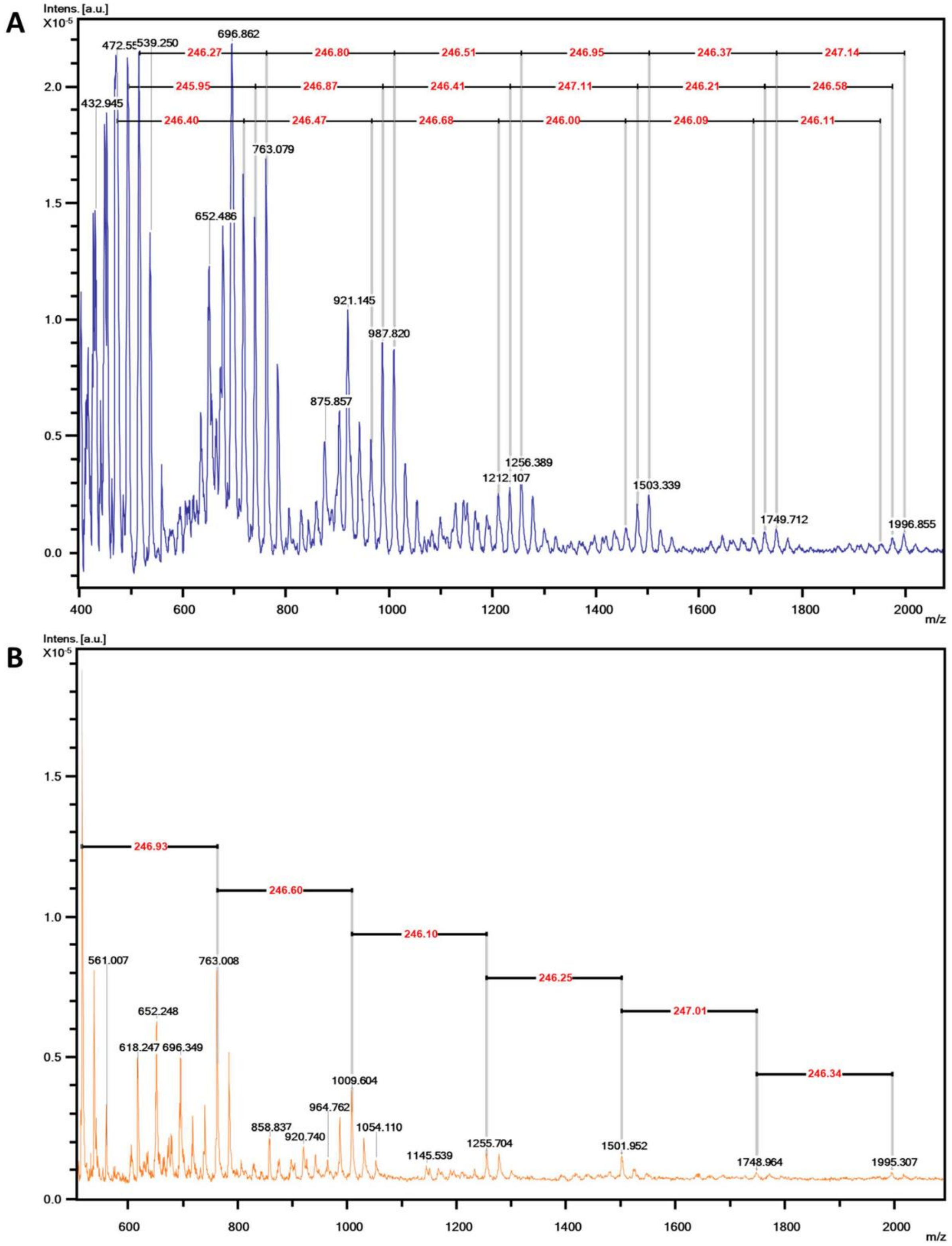

Figure S2. MALDI-TOF spectrum of $oDHF_{HW}$ prepared using SA (A) and 2,5-DHB (B) as a MALDI matrix material.

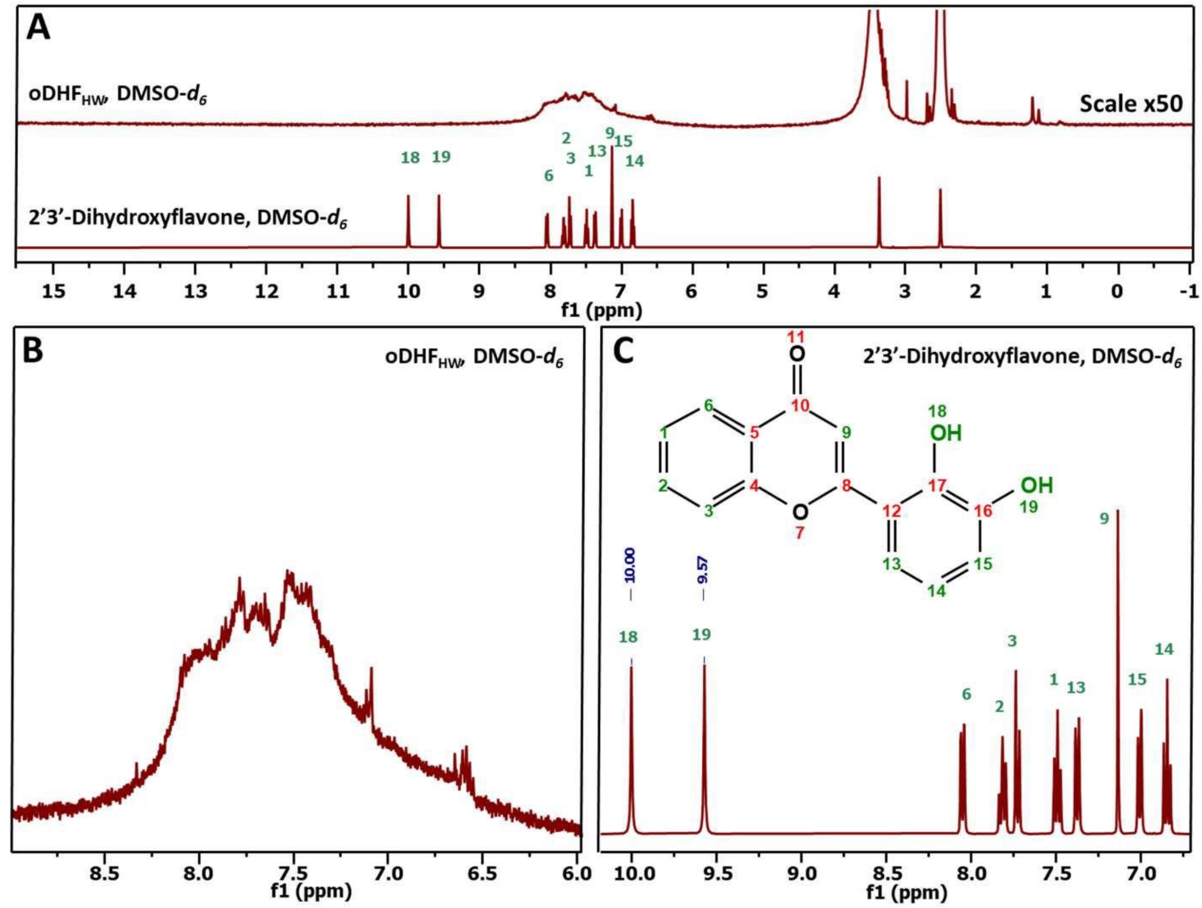

Figure S3. The ^{1}H NMR spectra of oDHF$_{HW}$ and 2'3'-dihydroxyflavone recorded in DMSO-d$_6$ (A). Aromatic region (9.0 - 6.0 ppm) of oDHF$_{HW}$ (B) and the zoomed spectra (10.5 – 6.5 ppm) of 2'3'-dihydroxyflavone aromatic and hydroxyl groups region.

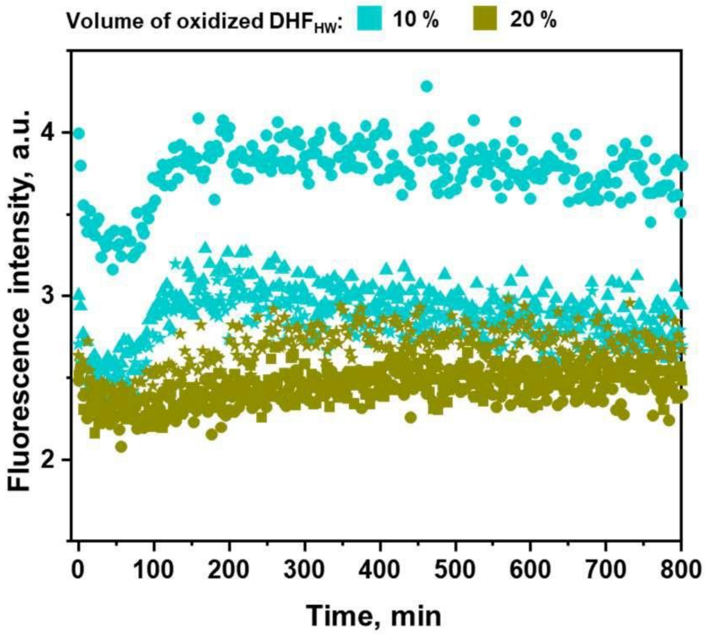

Figure S4. The kinetic curves of 1 μM Aβ aggregation with 10 % and 20 % of oDHF$_{HW}$.

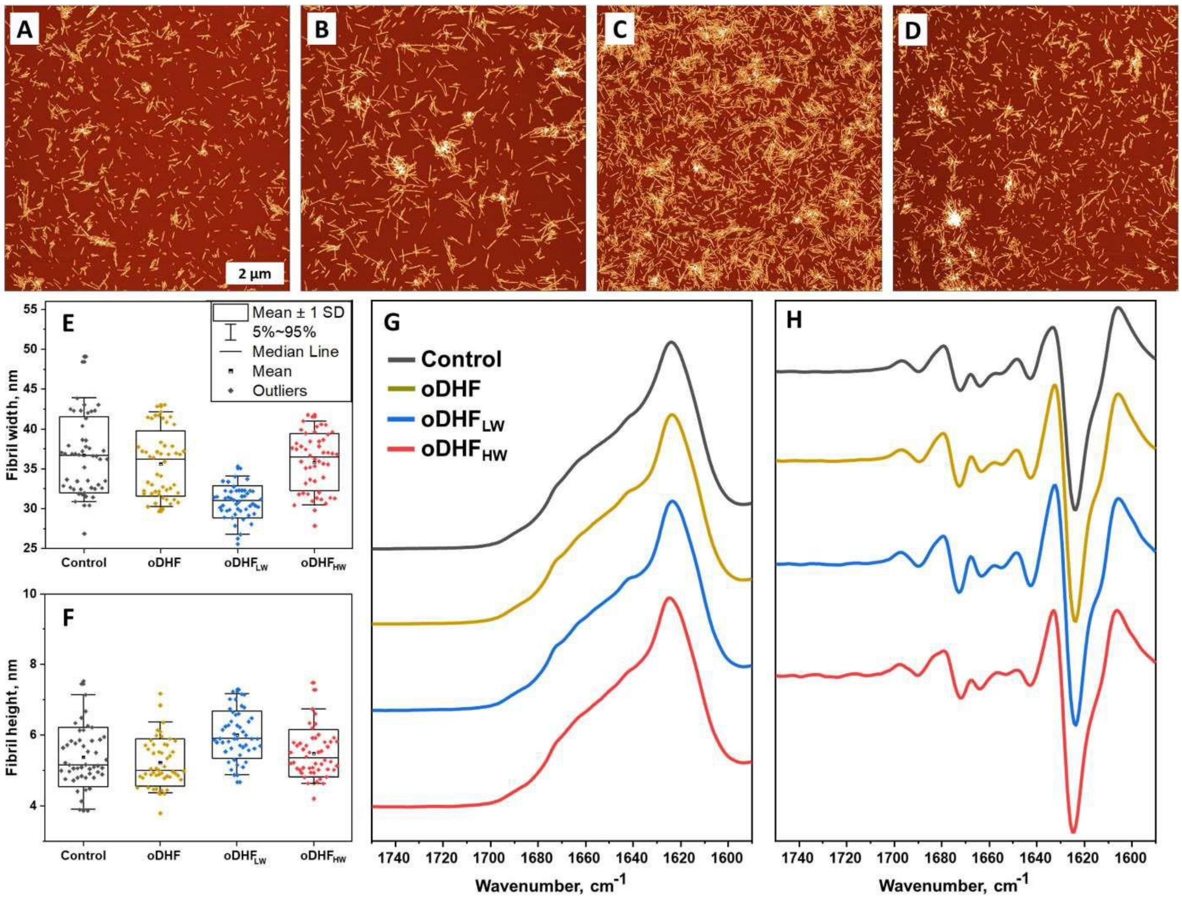

Figure S5. Atomic force microscopy (AFM) images of aSyn without (A) and with oDHF (B), $oDHF_{LW}$ (C) and $oDHF_{HW}$ (D). The fibril height (E) and width (F) distribution, where box plots indicate mean ± SD and error bars are in the 5% - 95% range (n = 50). The FTIR spectra of aSyn fibrils formed with or without inhibitors (G) and their second derivatives (H).
